# Enhancing Crowd Monitoring System Functionality through Data Fusion: Estimating Flow Rate from Wi-Fi Traces and Automated Counting System Data

**DOI:** 10.3390/s20216032

**Published:** 2020-10-23

**Authors:** Dorine C. Duives, Tim van Oijen, Serge P. Hoogendoorn

**Affiliations:** Department of Transport & Planning, Faculty of Civil Engineering & Geosciences, Delft University of Technology, Mekelweg 1, 2628 CD Delft, The Netherlands; t.p.vanoijen@tudelft.nl (T.v.O.); s.p.hoogendoorn@tudelft.nl (S.P.H.)

**Keywords:** crowd monitoring system, data fusion, Wi-Fi sensor data, automated counting systems, pedestrian movement dynamics, crowd management, RNN-LSTM, ARMAX, mass events

## Abstract

Crowd monitoring systems (CMSs) provide a state-of-the-art solution to manage crowds objectively. Most crowd monitoring systems feature one type of sensor, which severely limits the insights one can simultaneously gather regarding the crowd’s traffic state. Incorporating multiple functionally complementary sensor types is expensive. CMSs are needed that exploit data fusion opportunities to limit the number of (more expensive) sensors. This research estimates a data fusion algorithm to enhance the functionality of a CMS featuring Wi-Fi sensors by means of a small number of automated counting systems. Here, the goal is to estimate the pedestrian flow rate accurately based on real-time Wi-Fi traces at one sensor location, and historic flow rate and Wi-Fi trace information gathered at other sensor locations. Several data fusion models are estimated, amongst others, linear regression, shallow and recurrent neural networks, and Auto Regressive Moving Average (ARMAX) models. The data from the CMS of a large four-day music event was used to calibrate and validate the models. This study establishes that the RNN model best predicts the flow rate for this particular purpose. In addition, this research shows that model structures that incorporate information regarding the average current state of the area and the temporal variation in the Wi-Fi/count ratio perform best.

## 1. Introduction

Crowd monitoring systems (CMSs) provide a state-of-the-art solution to manage large crowds objectively. In recent years, researchers have discovered the potential of CMSs for crowd behavior research and have started to leverage CMSs to derive new insights regarding crowd movement behavior. For instance, ref. [1] inferred the pedestrian traffic state (i.e., walking velocity, density, and flow rate) at a nautical event (SAIL 2015) using a comprehensive CMS and ref. [2] determined activity patterns and destinations of staff and students on a university campus. Other researchers explored to what extent social media crawlers can be used as a tool to monitor activity schedules and movement behavior of crowds [3,4].

Most CMSs mentioned in literature feature only one type of sensors. An early bird in this respect is the use of RFID CMSs to track the progress of athletes in a marathon. In recent years, CMSs using Bluetooth and Wi-Fi sensors are commonly adopted to track activity patterns of individuals in urban environments [5,6] and CMSs featuring GSM cell-tower data are used to study aggregate (inter-city) movement patterns [7]. Yet, single-sensor sensing networks can produce a limited range of insights. In particular, Section 2 will show that most monitoring techniques either determine density, flow rate or route split information. Crowd managers generally require detailed insights featuring all three crowd movement characteristics simultaneously to manage large crowds. Thus, there is a need for CMSs that deliver a comprehensive estimate of the state of a crowd.

Multiple functionally complementary sensor types can be deployed in one sensor network in order to extend the functionality of CMSs, the potential of which was illustrated by ref. [1]. However, outfitting all sensor locations in a sensor network with multiple sensor systems is very expensive. In order to limit operation costs of CMSs, data fusion techniques are required that utilize the strengths of the overlapping presence of complementary sensor types in a limited number of locations in order to derive additional crowd state information at locations where only one sensor type is present, in general the least expensive one. 

This paper estimates a data fusion algorithm that enhances the capabilities of a CMS that is mainly equipped with Wi-Fi sensors. More specifically, this research establishes whether a network of Wi-Fi sensors can be used to predict pedestrian flow rates during a large music event by enhancing the CMS’s sensor network with a limited number of complementary automatic counting systems. Data from an operational CMS featuring a four-day music festival in the Netherlands is used to calibrate and validate the new algorithm.

Data fusion is defined by Hall and Llinar (1997) as the combination data from multiple sensors and related information from associated databases to achieve improved accuracy and more specific inferences than could be achieved by the use of a single sensor alone. In this study, various types of models are estimate to translate Wi-Fi counts to flow rate information. All models leverage data from multiple semantically distinct sensors (i.e., Wi-Fi and automated counting systems) in order to infer the flow rate for one particular sensor location. Yet, some more simple model types use the data from multiple data sources in the sensor network only to derive the model. During application, they will only accept the locally available Wi-Fi counts as an input. In contrast, other more sophisticated model types use the data from multiple data sources during application in order to improve the flow rate estimation at one specific location. The implementation of various model types allows us to quantify the potential improvement of the flow rate inferences as a result of real-time data fusion.

This paper will continue as follows. First, the research on monitoring systems for crowd management purposes is reviewed in Section 2. Afterward, the methodology to determine the best flow rate estimation model is presented in Section 3. This section introduces the technical details of the CMS’s sensors, the model types under investigation, and the goodness-of-fit metrics adopted to determine the best flow rate estimation model. Section 4 describes the case study and Section 5 presents the data used to estimate and validate the models. Afterward, the results of the case study are presented in Section 6. This paper concludes with a summary of the main findings and several suggestions for further research.

## 2. Overview of Sensor Systems for Crowd Monitoring Purposes

Multiple CMSs have been developed in recent years and many functionally distinct sensor types have been used to provide input to CMSs. Section 2.1 provides an overview of the most used sensor types in a CMS and discusses their (dis)advantages. This overview shows that the most comprehensive pedestrian traffic state estimation can be derived using a combination of Wi-Fi sensors and automatic counting systems. Accordingly, Section 2.2 zooms into the state-of-the-art featuring the usage of Wi-Fi sensor data for pedestrian traffic state estimation purposes.

### 2.1. Techniques to Monitor Crowd Movement Behavior

In general, six monitoring techniques exist that provide real-time information regarding the movements of crowds, being camera systems, automatic counting systems, RFID sensors, Wi-Fi/Bluetooth sensors, GPS sensors, and social media data. This section briefly discusses each of the monitoring techniques, including a description of the sensor type, recent examples of CMSs that make use of this monitoring technique and the type of insights that can be gathered regarding crowd movements using a network consisting solely of one sensors type.

One of the most popular CMSs is a system consisting of live camera feeds, for instance, closed-circuit television (CCTV). Ref. [8] mention the usage of an extensive network of CCTV systems to manage the Hajj in Mecca. The authors also have often encountered this type of CMS in the control centers of mass events in the Netherlands. Here, specialist security personnel that is trained to analyze crowd movements and to detect incidents monitor the live video feeds. A camera network provides crowd managers with qualitative insights regarding the crowd’s movements, for instance population characteristics and movement speed.

In combination with computer vision algorithms, live video feeds can also serve as a digital sensor that produces a quantitative output. For example, refs. [9,10] identify abnormal crowd movement behavior using optical flow algorithms. Ref. [11] determine the crowd’s density using the image texture. More recently, automated counting of pedestrians based on video feeds was introduced [12,13,14]. The insights regarding the crowd’s movements gathered using a camera or CCTV system predominantly pertain to the cameras’ field of view. Moreover, this CMS type requires an extensive support infrastructure (i.e., power and communication infrastructure), which can be difficult to arrange. Besides that, the installation of new surveillance systems is often controversial, given the increasing concerns regarding the right to be forgotten.

Recently, several other types of digital sensors have been introduced that can also count pedestrians, such as thermal cameras [15] and depth sensors [16,17]. The main advantage of these newer counting techniques intrinsically protects the privacy of pedestrians. However, require near top-down vantage point and continuous power supply, which is often challenging to realize in practice. Both requirements limit the widespread usage of these new sensing techniques for crowd monitoring at outdoor locations. They are, however, progressively used to monitor indoor venues, such as transfer hubs [18].

Active and passive Radio Frequency Identification (RFID) sensors are often used during sports events, the entertainment business and religious events [19,20,21]. As passive RFID sensors are relatively small, cheap and need no or very little power, they are easily incorporated into wearables that can be handed out to visitors, such as tickets, wristbands, and t-shirts. The identification information retrieved from passive RFID sensors is spatially sparse and dependent on the location of the sensing stations. Active RFID sensors emit a continuous signal, which ensures a spatially and temporally continuous signal of visitors’ locations. For long-term monitoring, often the sensing stations need to be hooked up to a power supply and communication infrastructure. Moreover, the range of RFID sensing stations is relatively small. 

Besides RFID, Wi-Fi sensors, which intercept communications signals transmitted via Wi-Fi and Bluetooth, can be used to monitor crowd movements. These near-field communication sensors capture the Media Access Control (MAC)-addresses, hereafter coined Wi-Fi trace, of the device that transmits the message, are more and more used to monitor crowd movements. Various researchers used Wi-Fi traces to identify pedestrian destination sequences [5,22], travel times [23] and arrival & departure times [24]. Wi-Fi sensors are quite small and can be connected to a small battery pack, which enables installation at locations where no or little electricity is available. A network of Wi-Fi sensors provides sparse spatial information regarding the location of a subset of the crowd. As Wi-Fi and Bluetooth sensors identify and record the MAC-address of Wi-Fi enabled devices in their vicinity, severe digital security measures are imperative to ensure the privacy of the public. Contrary to RFID sensing, Wi-Fi/Bluetooth sensing does not require actively involving the crowd. 

Another sensor type makes use of the Global Positioning System (GPS). A self-contained pocketsize GPS sensor and smartphone applications with an in-built tracking function are most often adopted for crowd monitoring purposes. Both GPS sensor types transmit, amongst other things, their GPS-position, speed and acceleration at regular time intervals. Literature illustrates that GPS trackers have been used to identify visitors’ routes, the walking speed and activity locations [1,25]. Similarly, smartphone applications have been adopted at the Hajj for crowd management purposes [26] and music events to track the traffic state [27,28]. Successful deployment of GPS sensors depends severely on the distribution strategy of the GPS trackers or smartphone application’s popularity, which varies between crowd types and contexts. Privacy concerns of GPS sensors are generally limited as users explicitly opt in to the tracking of their movements.

The last data type features social media crawlers, which analyze (geo-posted) messages from popular social media platforms, such as Twitter and Instagram. Social media crawling has been used to study mobility patterns [29], traffic anomalies [30] and the number of people in a restricted area [3,4,31], moreover, mapped demographic information, human movement patterns, and the popularity of Points of Interest (POIs). Social media are most often used to determine aggregate statistics of crowds. In general, the potential of social media crawling is very dependent on who shares what at what moment in time via which medium under what license. No privacy concerns arise when visitors pro-actively share their messages with the world.

Crowd managers require a range of insights regarding the crowd’s movements, among other things, atmosphere, anomalies, walking speed, density, flow rate, route choice, and destination choices. Here, density refers to the number of individuals that is present within a limited space at a certain moment in time (in P/m^2^) and the flow rate refers to the number of individuals that passes a certain cross-section within a limited amount of time (in P/m/s/). Table 1 presents a summary of the insights that, according to literature, can be derived using the monitoring techniques introduced above. Furthermore, the table identifies whether a part of the full population is monitored, whether the data is spatially discrete or continuous and whether privacy concerns can be expected. As one can see, none of the monitoring techniques can derive all the required insights. Moreover, none of the techniques can provide a comprehensive state estimation within an area featuring the entire population.

A combination of monitoring techniques is required to enhance the functionality of CMSs. The overview illustrates that several combinations of semantically distinct sensor types can provide complementary information. One of these combinations is the combination of automatic counting systems and Wi-Fi or RFID sensors. The benefits of the combination of these two static sensor types are that both sensor types are readily available in crowd management practice and do not rely on the active involvement of the crowd. Moreover, together they can potentially gather the most diverse set of insights pertaining to crowd’s state at discrete points in the network. Thus, this paper will specifically focus on the combination of automated counting systems and Wi-Fi sensors. 

### 2.2. Monitoring the Pedestrian Movement Behaviour Using Wi-Fi & Bluetooth Sensors

Wi-Fi and Bluetooth scanners are more and more readily adopted to analyze movement patterns in urban environments. These studies featured, amongst other things, highway traffic [32,33], public transit, pedestrian spaces in city centers [22,34], campuses [35,36], mass events [1,37,38] and buildings [39,40].

Table 2 presents a large, though not comprehensive, overview of the studies featuring the tracking of pedestrian movement patterns using Wi-Fi or Bluetooth sensors. The table illustrates that nearly all these studies use Wi-Fi sensors to analyze aggregate movement patterns in urban areas. Most frequently studies monitored the origin-destination patterns of pedestrians, the sequence of scanners that recorded a Wi-Fi trace (hereafter coined route), and the number of unique Wi-Fi traces identified by a sensor within a specific time period [34,41,42,43]). A limited set of studies details more intricate procedures to derive the time spent at a location and the travel time between sensors [23,44,45,46]. Some studies also attempted to identify the full trajectory of pedestrians using a network of Wi-Fi scanners and various types of triangulation algorithms [39,47,48].

A second set of studies has attempted to derive more detailed pedestrian traffic state information. Most of these studies determined the flow rate at a corridor or intersection over a relatively long time period [43,49,50,51,52]. Yet, to the authors’ knowledge, only ref. [53] identified the flow rate at a very precise temporal and spatial scale, namely pedestrians per meter per second. Meanwhile, little research has been performed concerning the derivation of walking speeds and crowd densities. For example, ref. [53] used several algorithms to determine the walking speed, and density of a crowd and ref. [37] translated the number of Wi-Fi enabled devices to the number of visitors near a Wi-Fi sensor. 

This overview illustrates that Wi-Fi sensors can be used to determine the trends in the traffic state variables density, walking speed, and flow. However, the translation from the number of Wi-Fi traces per interval to traffic state variables remains challenging, because the range of a Wi-Fi sensor is dependent on the crowd density [37]. Besides that, the detection rate of the Wi-Fi enabled devices is conditional to the type of pedestrian behaviors that are monitored. That is, the detection rate differs between moving and stationary pedestrians. 

Preliminary research of Duives et al. [37] illustrates that the combination of automatic counting systems and Wi-Fi sensors can be used to translate the number of traces to the actual number of pedestrians for a specific context (i.e., a stationary crowd near a music stage). However, this study also indicates that more research is needed to determine which model type can best translate Wi-Fi traces to flow rates in real-time for moving crowds. Moreover, this paper has not studied the potential of data fusion in order to improve the translation.

## 3. Introduction of the Data Format, Data Fusion Methods, and Goodness-of-Fit Metrics 

Multiple mathematical models can be used to infer flow rates from Wi-Fi sensors using automatic counting system data. As there is no predefined idea regarding the model type that would be best suited for this purpose, this research will compare several models with different mathematical structures, some of which apply data fusion in order to improve the flow rate estimation. In order to illustrate the differences between the various types of models, a mixture of straightforward and more sophisticated (data fusion) models is estimated. 

Section 3.2 will introduce all selected model types. Beforehand, Section 3.1 presents the format of data that serves as input and output of the data fusion model. The last Section 3.3 presents the goodness-of-fit metrics used to determine the best method. For a description of the case study and an introduction of the data, the reader is referred to Section 5.

### 3.1. Introduction Data Format

The sensor network of the case study produces two types of data, namely automated counts and Wi-Fi data. Every minute, the automated counting systems push a message to the server consisting of five fields, namely an identification code of the sensor, the timestamps at which the counting interval respectively starts $T_{begin}^{s}\left(t\right)$ and ends $T_{end}^{s}\left(t\right)$, and the total number of pedestrians that walked away from $N_{up}^{s}\left(t\right)$ and towards $N_{down}^{s}\left(t\right)$ the camera within the time interval (Equation (1)). The total flow rate per minute at a cross-section $q_{total}^{s}\left(t\right)$ is accordingly derived as the sum of the two flow rates (Equations (2) and (3)). The total flow rate is the only dependent variable in all estimated (data fusion) models.

(1) $$\langle id,T_{begin}^{s}\left(t\right),T_{end}^{s}\left(t\right),q_{up}^{s}\left(t\right),q_{down}^{s}\left(t\right)\rangle$$

(2) $$T^{s}\left(t\right)=T_{end}^{s}\left(t\right)-T_{begin}^{s}\left(t\right)$$

(3) $$q_{total}^{s}\left(t\right)=\frac{N_{up}^{s}\left(t\right)}{T^{s}\left(t\right)}+\frac{N_{down}^{s}\left(t\right)}{T^{s}\left(t\right)}$$

Wi-Fi sensors produce lists of hashed MAC-addresses. Each record of this list consists of at least the sensor id, the hashed MAC-address, the timestamp at which the Wi-Fi trace was first seen, the signal strength, and the device id. The list of active Wi-Fi enabled devices contains noise consisting of, for instance, stationary Wi-Fi enabled devices near the Wi-Fi sensor and Wi-Fi enabled-devices that transmit rotating MAC-addresses. In order to work with the list of MAC-addresses, first the list of MAC-addresses is filtered (see Table 3 for the procedure). 

The resulting list contains the hashed MAC-addresses and the timestamps at which each MAC-address was in range of a particular Wi-Fi sensor. The number of Wi-Fi enabled devices that are registered per minute is derived $N_{WiFi}^{s}$, hereafter identified as Wi-Fi count, which is the length of the cleaned list of MAC-addresses. The Wi-Fi count reflects the number of unique Wi-Fi enabled devices within the vicinity of the sensor, and is as such, a proxy for the pedestrian density in a pedestrian-only area.

Important to realize is that the Wi-Fi counts of different sensor locations cannot be added or subtracted as the same individual can pass multiple sensors during a one-minute time interval. Moreover, if multiple Wi-Fi sensors jointly cover one cross-section, the raw list of hashed MAC-addresses of both sensors should be combined first before starting the filtering and transformation procedure. The Wi-Fi count time series serves as the main input for all data fusion models that are estimated in this study. 

Some model types featured in this study accept additional contextual variables. For instance, one could add the data from all other sensors to the input of a model for one location. However, the resulting model would be very difficult to generalize, which severely limits the application of the estimated model. Thus, contrary to other data fusion endeavors, aggregate statistics featuring the overall status in the sensor network are used as additional inputs. In total, five additional input variables are created that provide more context to the Wi-Fi count time series, which are:

1. the time interval between the current time and the start of the event on each day ΔT,
2. the total Wi-Fi count in the sensor network at the current time step $\sum \;N_{wifi}\left(t\right)$,
3. the average total flow rate in the sensor network $\bar{\sum \;q_{tot}\left(t\right)}$,
4. the average difference between consecutive total flow rate measurements $\bar{\sum \;q_{tot}\left(t\right)-q_{tot}\left(t-1\right)}$,
5. the average multiplication factor $\bar{r}$(t).

Here, the average total flow rate and average difference in the total flow rate are calculated as a moving average over the last 5 min or 10 min. Earlier work on Wi-Fi sensors pointed out that these intervals produce relatively good results for dynamic flows [37]. The average multiplication factor is computed by dividing the total flow rate over the total Wi-Fi counts for a given day. Please note, that all data fusion models featured in this study are estimated using the same ‘base’ set of variables. The explanatory variables that are featured in the best model of a certain model type might differ as a result of the estimation procedure.

### 3.2. Introduction Data Fusion Methods

Many different data fusion models can be used to estimate flow rates using Wi-Fi trace data and counts. In order to determine which one is best suited, an array of models will be tested. Here, model types are selected that either were encountered by the authors while working with crowd management practitioners or are often adopted for time-series analyses. In particular, (in)direct approximation using linear, multiple linear regression models and shallow neural networks are often encountered in practice. RNN and ARMA models are often adopted in research for traffic time series prediction [64,65]. Besides that, for most model types a ‘simple’ prediction application as well as a more sophisticated data fusion application is derived. Here, prediction applications only accept the locally available Wi-Fi count as input, while the data fusion applications also accept exogenous variables constructed using real-time data from other sensors in the network.

Below, this section introduces the following flow rate prediction models: (1) + (2) an indirect and direct approximation using linear and multiple linear regression models, (3) a shallow neural network, (4) a recurrent neural network with a long short-term memory unit (RNN-LSTM) and (5) a Autoregressive Moving Average model (ARMAX). 

#### 3.2.1. Indirect (Multiple) Regression Models

In practice, often the global average ratio between the flow rate at a cross-section and the count of Wi-Fi enabled devices is determined using a two-step procedure. First, a multiplication factor is determined using the historic Wi-Fi counts and flow rate data. Accordingly, the multiplication factor is used to determine the current flow rate at a cross-section (see Equation (4)). Even though, this is indirect and inefficient procedure, the authors encounter this method often in practice.

(4) $$q_{total}^{s}\left(t\right)=r^{s}\left(t\right)*N_{WiFi}^{s}\left(t\right)$$

Given that the relation between the multiplication factor and the Wi-Fi counts is not directly apparent from the data, four distinct mathematical models are fitted, namely:

Model 1a. a linear model without a constant
  (5) $$r^{s}\left(t\right)=c_{2}N_{wifi}^{s}\left(t\right)$$
Model 1b. a linear model with constant
  (6) $$r^{s}\left(t\right)=c_{2}N_{wifi}^{s}\left(t\right)+c_{1}$$
Model 1c. a quadratic model with constant
  (7) $$r^{s}\left(t\right)=c_{3}N_{wifi}^{s}{\left(t\right)}^{2}+c_{2}N_{WiFi}^{s}\left(t\right)+c_{1}$$
Model 1d. a logarithmic model with constant
  (8) $$r^{s}\left(t\right)=c_{4}+\frac{c_{5}}{log\left(c_{6}N_{wifi}^{s}\left(t\right)\right)}$$

Here, $c_{x}$ represents the parameters of the regression model featuring a certain data type. All four models determine a global multiplication factor using the information from the locations where counting data is available. That is, the time series data from all sensor locations that are equipped with both sensor systems are used to determine one multiplication factor that will be used to estimate the flow rate at all sensor locations.

#### 3.2.2. Direct (Multiple) Regression Model

The use of an indirect approximation of the flow rate introduces additional (unwanted) noise. A model that directly relates the flow rate to the Wi-Fi counts circumvents this issue. Here, the variance in the data is directly incorporated in the model, which leads to slightly different model formulations and parameter settings in case of the more complex model specifications. Also in this case, the relation between the two variables is not clear-cut. Thus, four different regression models are fitted, namely:

Model 2a. a linear approximation without a constant
  (9) $$q_{total}^{s}\left(t\right)=c_{2,wifi}N_{wifi}^{s}\left(t\right)$$
Model 2b. a linear approximation with constant
  (10) $$q_{total}^{s}\left(t\right)=c_{2,wifi}N_{wifi}^{s}\left(t\right)+c_{1}$$
Model 2c. a quadratic approximation
  (11) $$q_{total}^{s}\left(t\right)=c_{3,wifi}N_{wifi}^{s}{\left(t\right)}^{2}+c_{2,wifi}N_{WiFi}^{s}\left(t\right)+c_{1}$$
Model 2d. an exponential approximation with constant
  (12) $$q_{total}^{s}\left(t\right)=c_{1}+c_{4,wifi}*exp\left(c_{5,wifi}N_{wifi}^{s}\left(t\right)\right)$$

Besides direct regression models with just one independent and one dependent variable, also exogenous variables can be added to the direct regression models. In this study, we have chosen to enhance only the linear regression model with additional contextual variables in order to limit the number of models that needed to be enumerated. The additional contextual variables that are presented in Section 3.1 are incorporated in the variable set. All subsets of the entire set of variables are tested. The best model is the model with the best model fit.

#### 3.2.3. Shallow Neural Network (NN)

Machine-learning techniques can also be used to fuse the Wi-Fi and flow rate data. Thus, also a shallow neural network is calibrated that features one layer of hidden nodes. This neural network can consider more information than the regression models. Thus, the additional contextual variables are incorporated in the shallow neural network. Depending on the number of extra variables that are considered, the shallow neural network has 2 to 6 input nodes. 

The best set of inputs and the number of hidden nodes are determined as part of the training. A forward selection model is applied, in which first a model without additional contextual variables is estimated. Accordingly, an additional model is estimated for all predictors that are not in the model, all of featuring one additional variable. Their adjusted coefficient of determination $R^{2}{}_{adj}$ are compared against the base model. Here, specifically the $R^{2}{}_{adj}$ is adopted in order to ensure the model that explains most of the overall variance is selected. Only models with the largest increase in the coefficient of determination (with a minimum of $R^{2}{}_{adj}>0.01$) is retained. In the following rounds, the retained model is enhanced step-by-step by adding variables using the same procedure until no more variables can be added. 

Given the nearly linear relation between the Wi-Fi counts and automated counts, the authors expect that a low number of hidden nodes will be sufficient. Thus, nets with a varying hidden layer size containing 1 to 10 hidden nodes have been trained. During training, the training data is split into 80% training data, 10% validation an 10% testing data. The training and test data samples are drawn at random from the training data set (Wednesday and Friday). As a result, the goodness-of-fit metrics can differ slightly between estimation runs. In order to account for the stochasticity, for each net size, 30 training runs are performed and the run with the 50th percentile of the coefficient of determination is used in the comparison between model fits. The best shallow neural networks with and without additional variables are presented in the results section.

#### 3.2.4. Autoregressive Moving Average (ARMAX) Model

The presentation of the flow rate and Wi-Fi count time series in Section 4 illustrates that the time series are stationary and autocorrelated. Therefore, also two model types are included that incorporate memory explicitly into the estimation process. The first type, i.e., the ARMAX model, explicitly takes into account state information from previous time. An ARMAX model can also accept more variables. Thus, similar to the neural network model, five additional contextual variables have been added to the model. The same model selection procedure has been adopted as for the shallow neural network model.

An analysis of the (partial) autocorrelation shows that the first ten lags are correlated. Therefore, ARMAX models with any combination of 1–10 autocorrelation lags and 1–10 moving average lags are estimated using the training data (i.e., Wednesday & Friday). The results for the best ARMAX models with and without additional variables are presented in the results section. 

#### 3.2.5. Recurrent Neural Network 

The second data fusion method, which formally incorporate memory, is a Recurrent Neural Network with a Long Short-term Memory (RNN-LSTM). In this type of network, each neuron keeps information content in memory and provides this information to the neuron as an additional input at the next time step. A RNN-LSTM is often used to estimate complex non-linear processes and processes in which autocorrelation exists. 

Similarly, to the shallow neural network, 2–6 input nodes are defined, one layer of hidden neurons containing 1–10 nodes and 1 output node (i.e., the flow rate). The input variables are similar to the inputs of the ARMAX model. The RNNs are trained using 80% training, 10% validation and 10% testing data, all of which featuring the training data set (Wednesday & Friday), using 10 runs per model specification and retaining the 50th percentile model fit. The best RNNs with and without additional variables are presented in the results section. 

### 3.3. Goodness-of-Fit Metrics

Two goodness-of-fit metrics are used to determine which data fusion method performs best, namely the adjusted coefficient of determination $R_{m,adj}^{2}$ (Equations (13)–(15)) and the Euclidian distance $RMSE_{m}$ (Equation (16)). The first metric is used to determine the fraction of the variance that is explained by the estimated models. A larger coefficient of determination represents a better model fit. The second metric is used to determine to what extent the trends in the time series are similar. The smaller the root mean square error, the more similar two time-series are.

(13) $$SS_{res}=\overset{T}{\underset{t=0}{\sum }}{\left(q_{tot,model}^{s}\left(t\right)-{\bar{q}}_{tot,data}^{s}\left(t\right)\right)}^{2}$$

(14) $$SS_{tot}=\sum_{t=0}^{T}{\left(q_{tot,data}^{s}\left(t\right)-{\bar{q}}_{tot,data}^{s}\left(t\right)\right)}^{2}$$

(15) Rm,adj2=1−SSres(n−p−1)SStot(n−1)

(16) $$RMSE_{m}=\sqrt{\sum_{t=1}^{T}\frac{{\left(q_{tot,data}^{s}\left(t\right)-{\tilde{q}}_{tot,model}^{s}\left(t\right)\right)}^{2}}{T}}$$

## 4. Introducing the TT Festival 2018

The data fusion methods are calibrated and validated using data from a real-life case study, namely data generated by the crowd monitoring system of the music and motorsport event TT Festival. The following section will first provide a general description of the event in Section 4.1. Afterwards, the sensing network is described in Section 4.2. The reader is referred to the following Section 5 for a presentation of the time series of the Wi-Fi counts and flow rates that were captured during the TT Festival.

### 4.1. The TT Festival

**Figure 1:**
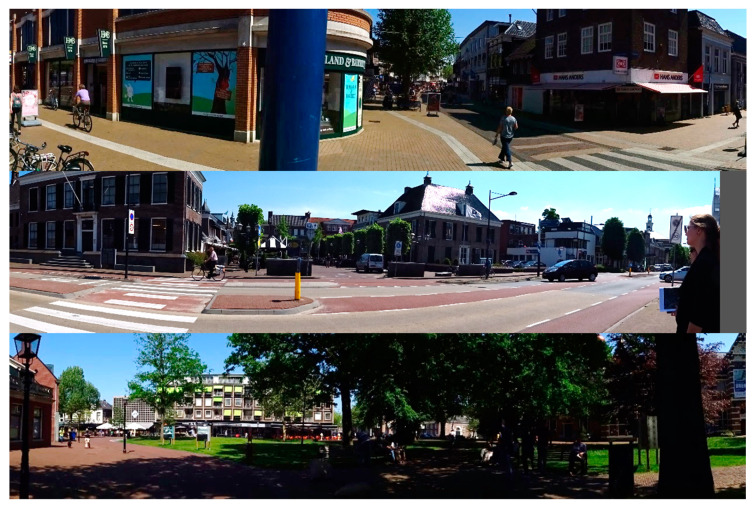

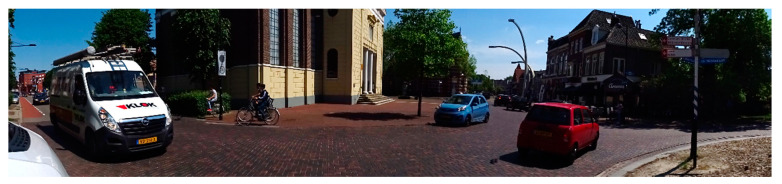
An impression of the city center of Assen, The Netherlands, f.t.t.b. Rolderstraat, de Markt, de Brink, Kerkplein.

Each year the TT Festival is held in Assen, The Netherlands, in concurrence with the MotoGP at the TT circuit nearby. In 2018, the TT Festival took place from Wednesday, 27 June, 18:00 until Sunday, 1 July, 04:00. During four consecutive evenings, activities were organized at 3 to 10 music stages in the city center of the municipality Assen. The inner city of Assen predominantly consists of two-to-four-story houses with retail and restaurants on the lower floors (see Figure 1). Streets are relatively wide according to Dutch standards and lined with trees. Most music stages were located on open squares and/or wide corridors.

A very mixed population of 20,000–65,000 visitors per day walked through the inner city of Assen during these four evenings. Depending on the time of the day, young children, elderly, teens, and adults were partaking in the entertainment. After midnight, especially teens and adults were present. Men and women equally took part in the festivities. Compared to other Dutch festivals, the average age of visitors is relatively high. Many visitors walked around in groups of friends or family. As the MotoGP is an internationally renowned event, many international MotoGP fans visit the TT Festival.

Each evening, the first acts commenced at 18:00 and the last acts finished between 01:00 and 04:00 at night. Each music stage had a specific theme and serviced a specific target audience. As the target audiences overlapped, visitors moved from stage to stage throughout the evening. During the opening hours of the TT Festival, the public spaces in the inner city were allocated to the visitors of the TT Festival. That is, cyclists and motorized vehicles were not allowed to enter the premises of the TT Festival during the opening hours of the event and pedestrians could safely use the entire width of the streets. 

### 4.2. Description of the Sensing Network

A crowd management center was set up to monitor and manage the crowds in the city center during the TT Festival. The daily operations mainly used CCTV in combination with crowd spotters to monitor the crowd’s movements. Besides that, a network of Wi-Fi sensors and several additional automated counting systems was installed to analyze the crowd’s movement objectively after the event finished. This analysis provided input for the layout and setup of the terrain in the future years. 

**Figure 2:**
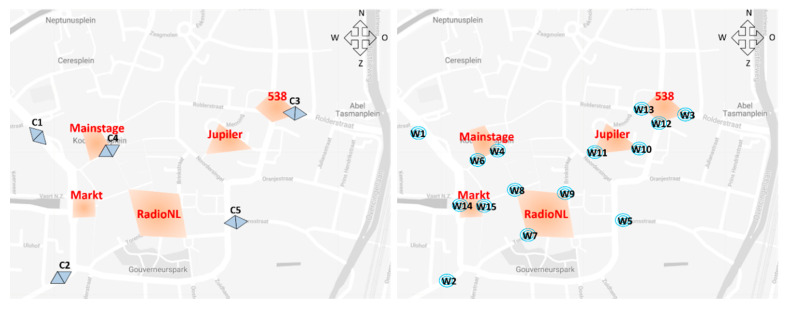
The layout of the sensor network depicted on a map for the TT Festival terrain of 2018, where the circles with the W-code represent the Wi-Fi sensors and the double triangles with the C-code represent the automated counting systems.

The sensor network was set up to derive three quantities, being flow rates at the entrances, stay duration times at the music stages and route splits. Automated counting systems were installed at the four main entrances (C1, C2, C3 and C5, see the sensor network displayed in Figure 2) to determine the first quantity. A fifth automatic counting system (C4) was deployed at a cross-section near the main stage for research purposes. The data from all five cameras are used in this research.

The latter two quantities are derived from Wi-Fi sensors, which were installed at all major intersections on the festival premises. As the city of Assen has quite an intricate street network, it was not possible to cover all intersections with Wi-Fi sensors. Therefore, Wi-Fi sensors were installed at intersections on the most direct routes between any two stages. 

## 5. Presenting the Wi-Fi Count and Flow Rate Time Series

The crowd monitoring system recorded data for the entire duration of the TT Festival, from Wednesday 27 June at 18:00 until Sunday 1 July at 06:00. Instead of using all the data captured during the festival, only the data of each day between 06:00 p.m. and 11:00 p.m. is used in this research. This action is performed in order to limit the measurement errors in the flow rate time series, which are known to increase after nightfall due to changing light conditions. Incorporating the effect of changing light conditions is identified as an opportunity for future research.

### 5.1. Time Series Wi-Fi Count and Flow Rate

**Figure 3:**
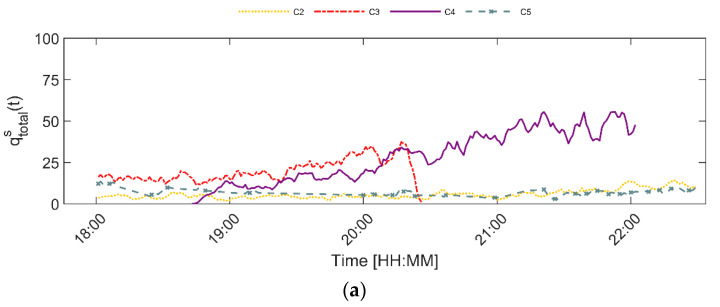

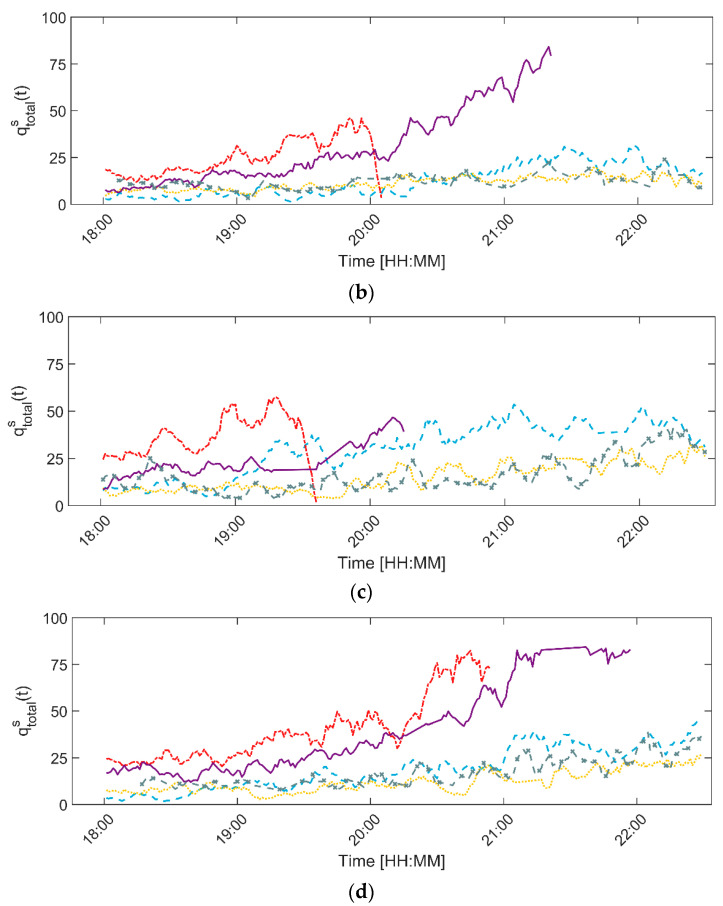
Flow rate time series per day and per location (c1–c5) for (**a**) Wednesday, (**b**) Thursday, (**c**) Friday and (**d**) Saturday.

The time series of the flow rate for each of the four days and five sensor locations are presented in Figure 3. These time series illustrate three trends. Firstly, a gradual increase in flow rate occurs on all days at all five sensor locations. During the time period visualized in the figure, more visitors are entering than leaving the premises. At the beginning of the event, a relatively gradual increase in the total flow rate is found. Later in the evening, the flow rate increases more steeply. Especially on Friday and Saturday, the inflow increases steeply between 20:00 and 22:00 at the counting systems C3 and C4, which are respectively located near the main entrance and main stage. Besides that, the time series also have several local maxima. These local fluctuations in the flow rate are most likely the result of the music programming. Visitors tend to move from stage to stage while stages are changing between acts. Consequently, specifically at the start and end of a performance, many visitors are on the move. Lastly, not all time-series are complete. During the TT festival the battery of two counting systems, i.e., camera C3 and C2, drained too quickly. Only the part of these two time series for which data is available are taken into account in the research.

**Figure 4:**
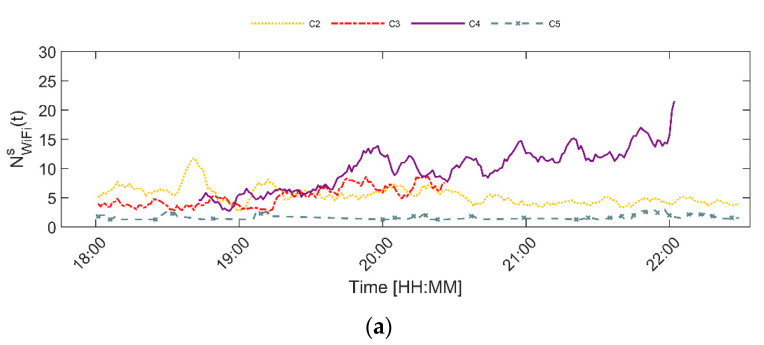

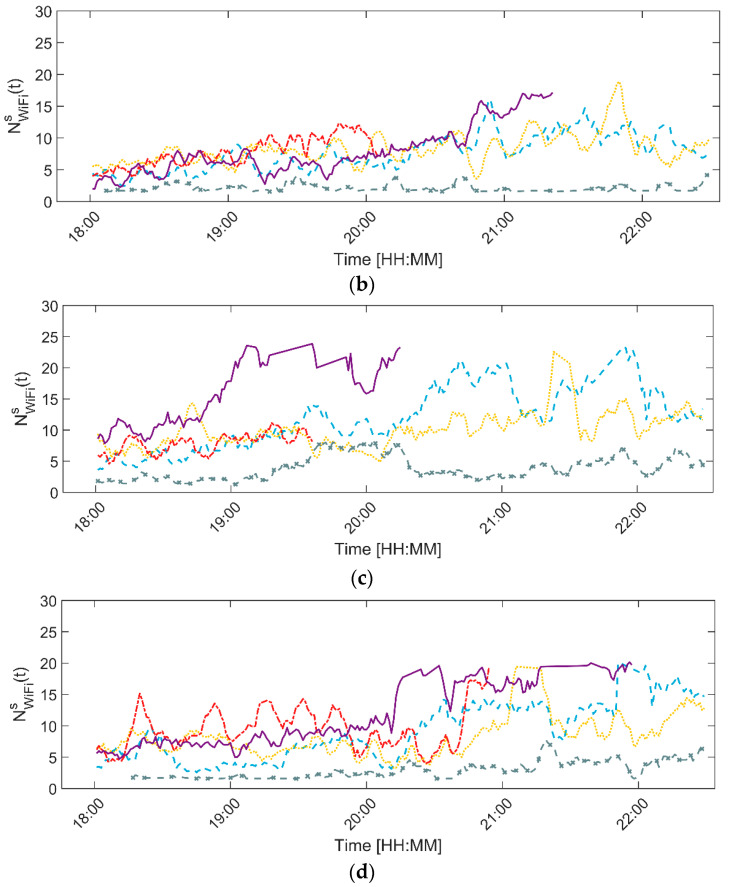
Time series Wi-Fi counts per day and per location (c1–c5) for (**a**) Wednesday, (**b**) Thursday, (**c**) Friday and (**d**) Saturday.

The time series of the Wi-Fi count, which are visualized in Figure 4, show different global trends than the time series of the flow rate. For instance, the Wi-Fi counts and flow rates at locations 2 and 5 differ a lot. That is, the Wi-Fi count of location 2 is consequently higher than the Wi-Fi count at location 5, while the flow rate time series of both locations are quite similar. In addition, the Wi-Fi count time series for location 1 has two large peaks on Friday, while the flow rate time series of the same day and location has four smaller peaks. 

Besides that, Figure 3 and Figure 4 illustrate that the time series of the flow rate on Wednesday and Thursday follow a similar trend. Equivalently, the flow rate time series on Friday and Saturday are comparable. Therefore, the time series from Wednesday and Friday are used for training purposes, while the time series captured on Thursday and Saturday are only used for validation purposes. This means, that the data from these two days has not been used to train any of the models presented underneath. Moreover, the data from the automated counting sensor for which the flow rate is estimated is not included in the aggregate statistics that serve as the additional contextual variables for that specific location. Consequently, the ground truth for a particular sensor location is the data that was derived on that particular day. The goodness-of-fit metrics displayed in the results section relate only to the validation data (Thursday and Saturday).

A student t-test confirms that the distribution of the values in the test time series of the flow rate is similar to the distribution of the values for the flow rate in the training time series. Thus, this split between training and validation data should not result in bias because of difference in the overall flow rate dynamics. 

### 5.2. Relation Between Wi-Fi Count and Flow Rate

**Figure 5:**
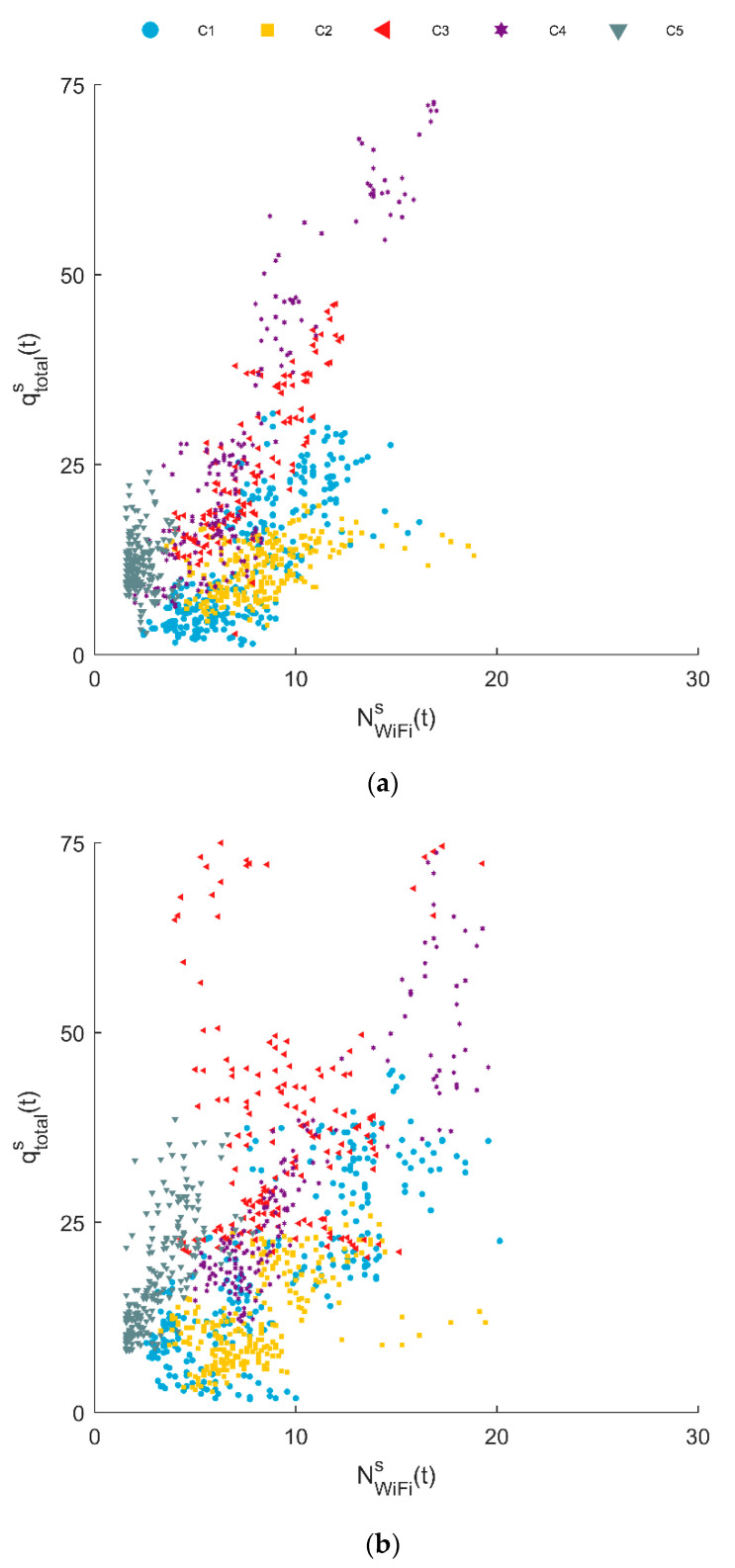
The relation between the Wi-Fi counts and the flow rate at sensor locations C1–C5, for (**a**) Thursday and (**b**) Saturday.

Figure 5 visualizes the relation between the automatic counts and the Wi-Fi counts. A positive relation between the two time-series can be established. However, the point cloud is very scattered, with quite some outliers in the upper left and lower right corner. The first type of outliers, with high flow rates and low Wi-Fi counts, is the result of the double identification of pedestrians are stationary within the camera’s field of view. The automated counting system to which these data points belong, i.e., C3, is located at a corridor where visitors tend to stand still to watch the attractions on both sides of the street. The second type of outliers, concerning low flow rates and high Wi-Fi counts, are the result of rare moments that all devices that are registered are by chance devices without a rotating MAC-address. 

Figure 5, moreover, illustrates that the point clouds of the different cameras do not entirely overlap. Consequently, the ratio between the flow rate and the Wi-Fi counts is expected to differ slightly between sensor locations. However, as the signature of the sensor location cannot be established for the sensor locations where only Wi-Fi sensors are installed, the differences between the sensor locations are not discounted in this research. Future research is needed to establish whether similarities in the Wi-Fi count signature or sensing setting layouts can be used to improve the flow rate prediction model further.

## 6. Best Data Fusion Model and Discussion of the Modeling Results

The data fusion methods introduced in Section 4 have been estimated using the training time series presented in Section 5. These models are tested using the test time series, i.e., Thursday and Saturday. Table 4 presents a summary of the goodness-of-fit metrics for all estimated data fusion methods. The three best models, which are those with the highest $R^{2}{}_{adj}$ and lowest RMSE, are an RNN-LSTM with one layer of 1 hidden node, a NN with 3 hidden nodes and a multiple linear regression model featuring all five additional contextual variables. All three models incorporate memory of previous time steps through the additional input variables or model structure.

In particular, the average total flow rate per sensor seems to be an essential additional determinant to estimate the flow rate at other locations. The presence of this variable in the best model indicates that the total flow rate influences the ratio between the Wi-Fi counts and the flow rate, which is in line with the results of ref. [37]. They established that the ratio between Wi-Fi sensors and the total number of static people near a sensor is dependent on the total number of people that are counted. That is, the more devices are detected, the lower the multiplication factor becomes. The second variable added to the RNN-LSTM is the average ratio between the Wi-Fi counts and flow rate. This finding suggests that A) the ratio between Wi-Fi counts and the flow ratio varies over time and B) this dynamic variance should be captured in order to predict the flow rate correctly. There can be multiple sources of the fluctuation of the multiplication factor. The authors expect that two important causes of this fluctuation in the multiplication factor are changes in the population mixture (i.e., the average age, and thus the type of smartphones changes during the evening) and changes in the walking speed (i.e., during the evening, the average walking speed was found to decrease).

**Figure 6:**
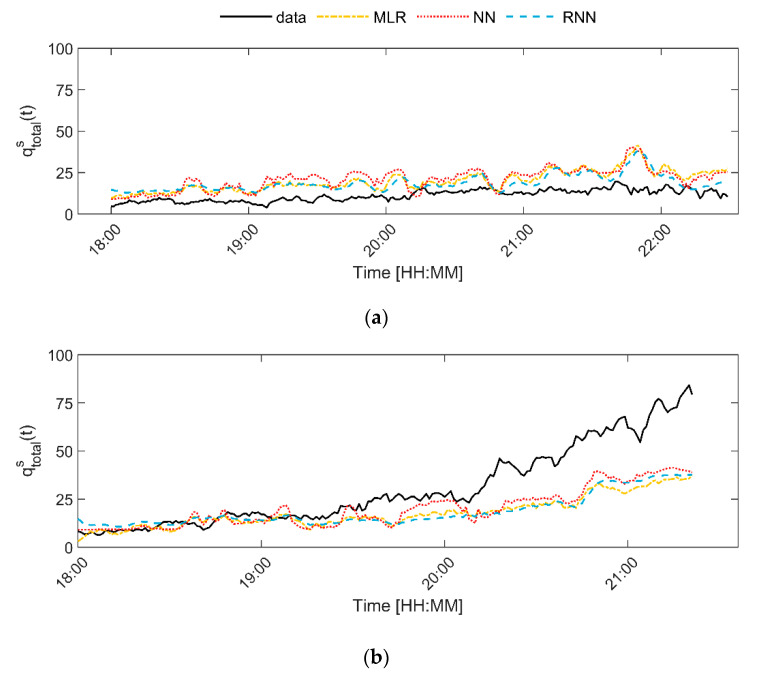

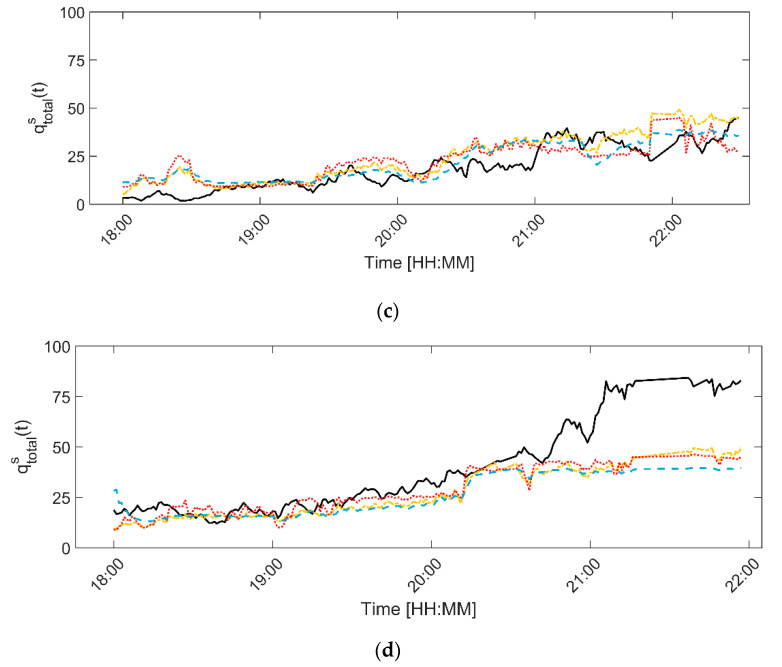
Comparison of simulated and measured flow rate time series for several locations and days, where (**a**) locations 2—Thursday, (**b**) location 4—Thursday, (**c**) location 2—Saturday, and (**d**) location 4—Saturday.

In order to get more insights into the forecasting capabilities of the models, the simulated flow rate time series are compared with the actually measured time series of the flow rate, see Figure 6 for a visual comparison of the results. The figure illustrates that the three models have slightly different characteristics. In general, the three best models (i.e., model 6—multiple linear regression, 8—shallow neural network—and 9—RNN-LSTM) are capable of predicting the general trend in the flow rate at times when the flow rate is limited, i.e., less than 25 visitors per minute. However, none of the models predicts the sharp increase in the flow rate that was measured at location 4 on Thursday and Saturday. This was also not expected, as the time series of the Wi-Fi count at this location does not provide any indication that a sudden change in flow rate is occurring. 

Furthermore, all model types have great difficulty capturing the exact measured values. This suggests that the translation between Wi-Fi counts and flow rate is not as direct as one would expect based on literature and, in particular, the pedestrian fundamental diagram [66]. The authors assume that this finding is the result of the event settings for which these models are estimated. In those settings, a pedestrian space serves multiple purposes simultaneously. That is, pedestrians use the same space to travel from A to B or to watch a performance. Depending on the mixture of these two behaviors, the expected relation between the Wi-Fi counts and flow rate differs. Future research into the unravelling of these two activity types might improve the flow rate estimation. 

Besides that, the several simple linear regression model (model 6) and the shallow neural network model (models 8) predict several strange peaks in the flow rate at moments in time that these do not occur in reality. These peaks are also found in the raw Wi-Fi counts, for instance between 18:00 and 19:00 at location 2. The lack of strange peaks in the output of the RNN-LSTM suggests that the RNN-LSTM dampens these peaks. This dampening effect is possibly why the coefficient of determination of the RNN is slightly higher than that of the other two models.

In addition, Figure 6 shows that the RNN model best captures the global trend at lower flow rates. Given that low flow rates occur most of the time, models, which most accurately predict low flow rates, are favored in the calibration process. Thus, the RNN is best capable of capturing both low and high flow rate values. 

**Figure 7:**
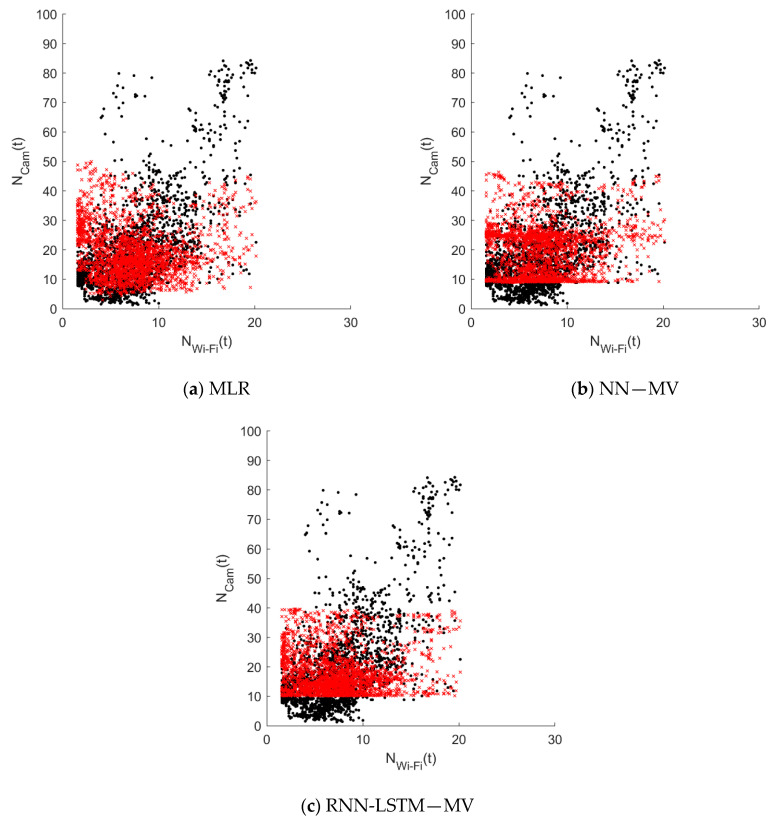
The relation between the ground truth (black dot) and the model estimation (red cross) for both validation days (Thursday & Saturday) and all sensor locations (i.e., C1–C5) for (**a**) model 6: MLN (**b**) model 8: NN—MV, and (**c**) model 9: RNN-LSTM—MV.

**Figure 8:**
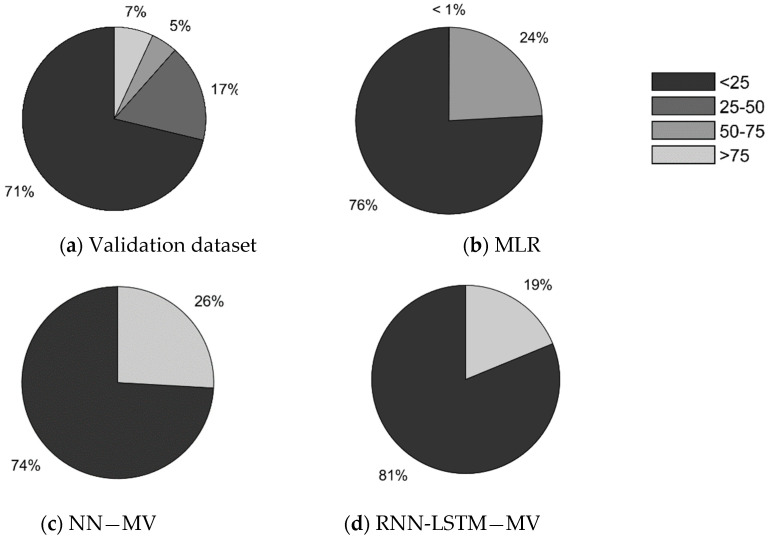
Pie charts comparing the percentage of measurements of a certain size within the validation dataset, where the validation dataset is depicted in (**a**) and the simulation outputs of model 6, model 8 and model 9 are depicted in (**b**–**d**).

A comparison of the scatter plots of the real data with the models’ results illustrates that all three models have difficulties predicting high flow rates correctly (see Figure 7). This is irrespective of the size of the Wi-Fi count. In all three graphs, distinct horizontal boundaries can be distinguished. An classification of the validation set into four bins also shows that the NN and RNN models do not generate any values higher than 50 (see Figure 8). The MLR model only generates a very low number of values higher than 50 (i.e., < 1%). In comparison with the validation dataset (15%), the complete lack of high value estimates by the models is striking. All three models are specialized in predicting the low flow situations correctly. Consequently, the three ‘best’ models cannot be used to identify high flow rate situations using Wi-Fi counts.

**Figure 9:**
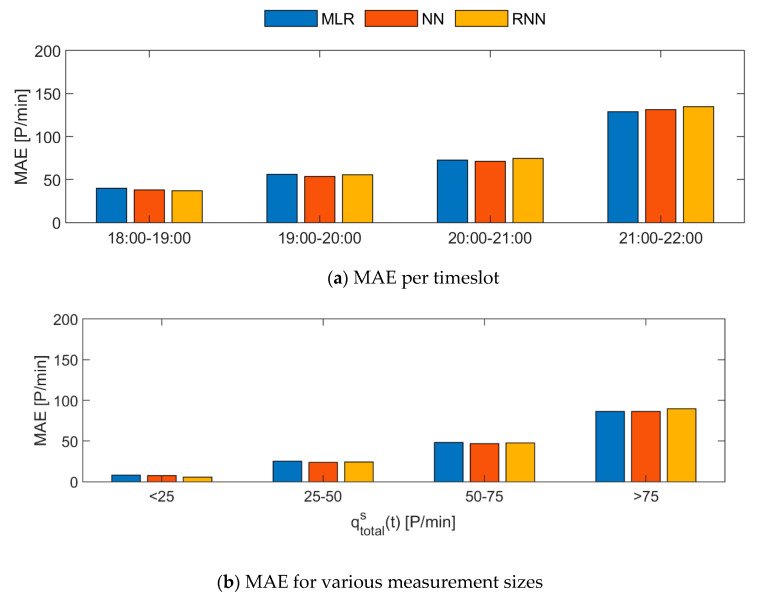
Bar graphs comparing the Mean Absolute Error (MAE) of the models’ results for the validation dataset for various timeslots (**a**) and sizes of the measurement (**b**).

A last analysis of the Mean Absolute Error (MAE) indicates that the MAE grows with time during the TT Festival (see Figure 9). The increasing f can partly be explained by the fact that the MAE for larger flow rates is bigger than for smaller flow rates. Since visitor numbers increase during the evening, higher flows occur at the end of the evening. However, the MAE between 21:00 and 22:00 is even larger than one would expect as a result of the higher flow rate. The authors expect that the very high MAE can also be partly explained by the changes in the visitor population. That is, more young people with state-of-the-art smartphones often results in a lower number of Wi-Fi traces as smartphones with rotating MAC-addresses are filtered out. Consequently, the three best models seem to be sensitive to the characteristics of the population. More research is required to, either explicitly incorporate the population characteristics in the models, or derive data fusion models that are in-sensitive to the population characteristics.

## 7. Conclusions and Future Works

Many crowd monitoring systems make use of only one type of sensor, which severely limits the type of insights regarding the crowd’s movements that one can gather simultaneously. This research has estimated a data fusion model that enhances crowd monitoring systems that predominantly feature one sensor type, namely Wi-Fi sensors. Using a small number of sensors of another type, namely automatic counting systems, the flow rate can be estimated for sensor locations where only Wi-Fi data is gathered.

Using historic data featuring the flow rate and Wi-Fi counts per minute, several data fusion models have been calibrated. Among others, various regression models, shallow neural networks, recurrent neural networks, and ARMAX models were estimated. The best three performing model structures are the Recurrent Neural Network (RNN-LSTM), shallow neural network (NN) and a multiple linear regression model. The NN estimated the flow rate the best, which is probably due to its ability to predict non-linear behavior.

By doing so, this research shows that the installation of a limited number of highly specialized sensors in a vast sensor network consisting of only Wi-Fi sensors can enhance the insights crowd managers can gather regarding the pedestrian traffic state. More specifically, this work illustrates that the installation of several well-placed automatic camera sensors allows one to approximate the flow rate at parts of a sensor-network where no automated counting systems are available.

Moreover, this research shows that feeding the MLR, NN and RNN-LSTM with additional variable pertaining the overall crowd movement dynamics at the terrain improves the flow rate estimates. This suggests that the sensor data is to some extent spatially correlated. Consequently, when deriving traffic state variables for one location, one can leverage the spatial and temporal correlations of other sensors in the network to improve the estimation. 

This work also identifies that the prediction technique has some limitations, which provide exciting alleys for further research. First of all, the current flow rate estimation model is not specialized per sensor location, as it is unclear to what extent the characteristics of the sensor location are influencing the ratio between the Wi-Fi counts and flow rate under different circumstances. A comparison of the Wi-Fi count and flow rate time series shows that differences exist between sensor locations. Therefore, a classification of similar sensor locations could potentially improve the flow rate estimation model. 

Moreover, a limited set of model structures is tested for the neural networks (both shallow and recurrent). As such, currently this study cannot be ensured that the current structures of these neural networks are the optimal structures. Potentially, deeper learning networks might further improve the models’ capabilities. More research is required to determine the optimal model structure of neural networks for this particular data fusion task.

Besides that, the current model is estimated based on one particular population of festival visitors. This diverse and relatively old population is not necessarily representative for other festivals. The authors expect that the type of populations might influence the validity of the data fusion model as smartphone usage might differ between population types. Consequently, the current flow rate estimation model cannot directly be used for other types of populations (e.g. pop concert visitors), or other settings (e.g. shopping malls or theme parks). Research into the influence of the population type and context on the model fit is required.

Last of all, the applicability of this method is dependent on the size of the population and the percentage of visitors that carries a Wi-Fi enabled device that transmits a non-rotating MAC-address whenever it is used. As the privacy-protocols installed on smartphones improve, the current filtering procedure becomes increasingly problematic. Therefore, further research into data fusion methods that can handle with noisy Wi-Fi data is essential.

## Figures and Tables

**Table 1:** Summary of insights that can be directly captured using a specific type of sensor without assumptions or information from other data sources.

Sensor Type	Variables							Monitored Crowd	Data Type	Privacy Concerns
	AT	AN	V	K	Q	RC	D			
Camera Systems	X	X	-	-	-	-	-	E	Point	Yes
Automated Counting Systems (Computer Vision Algorithms)	-	-	X	X	X	-	-	E	Point	Yes
Automated Counting Systems (Depth Sensor/Laser/IR)	-	-	X	X	X	-	-	E	Point	No
Passive RFID Sensors	-	-	-	-	-	X	X	S	Point	No
Active RFID Sensors	-	-	-	-	-	X	X	S	Point/area	No
Wi-Fi / Bluetooth Sensors	-	-	-	-	-	X	X	S	Point	Yes
GPS Tracker	-	-	X	-	-	X	X	S	Area	No
GPS Smartphone Application	-	-	X	-	-	X	X	S	Area	Yes
Social Media (text)	X	X	-	-	-	X	X	S	Area	No
Social Media (image)	X	X	-	X	-	X	X	S	Area	No

AT = atmosphere, AN = anomalies, V = walking speed, K = density, Q = flowrate, RC = route choice, D = destination, S = subset of the population, E = entire population.

**Table 2:** Filtering procedure Wi-Fi sensor list.

Combine records with distinct ids but similar device ids (i.e., hashed and shortened MAC-addresses). Delete records of device ids that only occur once in the list. Delete records of device ids that are only found at one sensor-location. Retain the first registration of ids at a sensor. For all sensor locations, retain following registrations of ids at a sensor if: the time between the first registration and the next is longer than 5 min and the id has been registered at another sensor in between.

**Table 3:** Summary of literature related to derivation traffic state using Wi-Fi & Bluetooth sensors.

Reference	Ref. No.	Mode	Setting	DL	Monitored Variables								
					Speed	Density	Flow Rate	TS	TT	Traj.	Route	OD	Presence
Chen et al. (2005)	[41]	P	Building	L	-	-	-	-	-	-	-	-	X
Miyaki et al. (2007)b	[54]	P	Outdoor pedestrian space	L	-	-	-	-	-	X	-	-	-
O’Neill et al. (2006)	[55]	P	City streets of Bath	L	-	-	X	X	-	-	-	-	X
Miyaki et al. (2007)a	[47]	P	City streets and Uni. of Tokyo	L	-	-	-	-	-	X	-	-	-
Millonig et al. (2008)	[56]	P	Mall	L	-	-	-	-	-	-	X	-	-
Bullock et al. (2010)	[44]	P	Airport security	H	-	-	-	-	X	-	-	-	X
Vu et al. (2010)	[57]	P	University Campus	L	-	-	-	-	-	-	-	-	X
Stange et al. (2011)	[42]	P	Racing event	H	-	-	-	-	-	-	X	-	X
Utsch et al. (2012)	[58]	P	Soccer stadium Nimes, France	H	-	-	-	X	-	-	X	-	-
Musa et al. (2012)	[22]	P	City streets of Chicago	L	-	-	-	-	-	-	X	-	-
Malinovskiy et al. (2012)	[45]	P	Unknown	L	-	-	-	-	X	-	-	-	-
Versichele et al. (2012)	[50]	P	Music event Ghent, Belgium	H	-	-	X	-	-	-	-	X	X
Abedi et al. (2013)	[59]	P, B	Grassy field	L	-	-	-	-	X	-	-	-	X
Bonne et al. (2013)	[38]	P	Mass event Pukkelpop, Belgium	H+L	-	-	-	-	-	-	X	-	-
Kostakos et al. (2013)	[49]	P	City streets of Oulu, Finland	L	-	-	X	-	-	-	-	-	X
Nawaz et al. (2013)	[60]	C	Parking lot	L	-	-	-	-	-	-	-	-	X
Xu et al. (2013)	[48]	P	City streets of Sydney	L	-	-	X	-	-	X	-	-	X
Danalet et al. (2014)	[5]	P	Campus	L	-	-	-	-	-	-	-	X	-
Fukuzaki et al. (2014)	[40]	P	Active Lab, building	L	-	-	-	-	-	-	X	-	X
Schauer et al. (2014)	[43]	P	Security gates airport	H	-	-	X	-	-	-	-	-	X
Fukuzaki et al. (2015)	[61]	P	Shopping mall	L	-	-	-	-	-	-	-	-	X
Farooq et al. (2015)	[46]	P	Festival, Montreal, Canada	H	-	-	-	-	X	-	-	X	X
Ma et al. (2015)	[52]	P	University building	L	-	-	X	X	-	-	-	-	X
Hoogendoorn et al. (2016)	[23]	P	Nautical event	H	-	-	-	-	X	-	-	-	-
Daamen et al. (2016)	[1]	P	Nautical event	H	X	X	X	-	X	-	-	-	-
Poucin et al. (2016)	[35]	P	Campus Concordia Uni. Montreal	L	-	-	-	-	-	-	X	-	X
Alessandrini et al. (2017)	[62]	P	Joint Research Centre (JRC) Ispra	L	-	-	X	X	-	X	-	-	X
Guo et al. (2017)	[63]	P	Building	P	X	-	-	-	-	-	-	-	X
Bellini et al. (2017)	[34]	P	City streets San Francisco	L	-	-	X	-	-	-	-	-	X
Fang et al. (2017)	[36]	P	University campus Dartmouth	L	-	-	-	-	-	-	X	-	-
Duives et al. (2018)	[28]	P	Music Event	H	-	X	-	-	-	-	-	-	-
Potortì et al. (2018)	[39]	P	Building	L	-	-	-	-	-	X	-	-	-

P = pedestrian, B = bicycle, C = car, L = normal city traffic, H = crowded pedestrian places, TS = Time spend, TT = Travel Time, Traj. = trajectory, OD = OD matrix.

**Table 4:** Summary of model estimation results, where the bold model is the best model. For all models, the ${\bar{R}}^{2}$ and RMSE are depicted for the Thursday and Saturday time series.

**Wi-Fi Counts Only**								
**Model No.**	**Model Description**						${\bar{R}}^{2}$	**RMSE**
1a	Indirect—linear without constant						0.1740	14.20
1b	Indirect—linear with constant						0.3590	12.51
1c	Indirect—quadratic						0.3590	12.51
1d	Indirect—logarithmic						0.3195	12.89
2a	Direct—linear without constant						0.3337	12.76
2b	Direct—linear with constant						0.3398	12.70
2c	Direct—quadratic						0.3590	12.51
2d	Direct—exponential						0.3591	12.51
3	ARMA (4,0,5)						0.1808	14.29
4	NN—2 nodes						0.3619	12.44
5	RNN—6 nodes						0.3582	12.478
**Wi-Fi counts + contextual variables**								
**Model No.**	**Model Description**	ΔT	$\sum N_{WiFi}$	$\bar{\sum q_{tot}}\left(t\right)$	$\bar{\Delta \sum q_{tot}}\left(t\right)$	$\bar{r}\left(t\right)$	${\bar{R}}^{2}$	**RMSE**
6	Direct—linear with constant	x	x	x	x	x	0.3849	12.24
7	ARMAX (3,0,3)	-	x	-	-	x	0.1167	15.54
8	NN—3 nodes	-	-	x	-	-	0.4306	11.77
9	RNN—1 node	-	-		-	-	0.3626	12.47
